# D-Glucose-Mediated Gold Nanoparticle Fabrication for Colorimetric Detection of Foodborne Pathogens

**DOI:** 10.3390/bios14060284

**Published:** 2024-06-01

**Authors:** Seo Yeon Park, Rajamanickam Sivakumar, Nae Yoon Lee

**Affiliations:** Department of BioNano Technology, Gachon University, 1342 Seongnam-daero, Sujeong-gu, Seongnam-si 13120, Gyeonggi-do, Republic of Korea; sayo2092@naver.com (S.Y.P.); sivachem1379@gmail.com (R.S.)

**Keywords:** D-glucose, gold nanoparticle, LAMP, foodborne pathogens, point-of-care testing, colorimetry

## Abstract

Gold nanoparticle (AuNP) fabrication via the oxidation of D-glucose is applied for detecting two foodborne pathogens, *Enterococcus faecium* (*E. faecium*) and *Staphylococcus aureus* (*S. aureus*). D-glucose is used as a reducing agent due to its oxidation to gluconic acid by sodium hydroxide (NaOH), resulting in the formation of AuNPs. Based on this mechanism, we develop AuNP-based colorimetric detection in conjunction with loop-mediated isothermal amplification (LAMP) for accurately identifying the infectious bacteria. Here, Au^+^ ions bind to the base of double-stranded DNA. In the presence of D-glucose and NaOH, the LAMP amplicon-Au^+^ complex maintains its bound state at 65 °C for 10 min while it is reduced to AuNPs in a dispersed form, exhibiting a red color. We aimed to pre-mix D-glucose with LAMP reagents before amplification and induce successful colorimetry without inhibiting amplification to simplify the experimental process and decrease the reaction time. Therefore, the entire process, including LAMP and colorimetric detection, is accomplished in approximately 1 h. The limit of detection of *E. faecium* and *S. aureus* is confirmed using the introduced method as 10^1^ CFU/mL and 100 fg/μL, respectively. We expect that colorimetric detection using D-glucose-mediated AuNP synthesis offers an application for simple and immediate molecular diagnosis.

## 1. Introduction

Rapid and specific analyses for the detection of foodborne pathogens are required owing to early diagnosis of infectious diseases. In particular, Gram-positive bacteria are opportunistic foodborne pathogens that need effective and novel treatment strategies because of their resistance to various antibiotics [1,2]. Their formidable resistance capabilities pose lethal challenges to hospitalized patients, underscoring the importance of rapid detection for prompt diagnosis and adequate treatment [3,4]. To find new remedies for these pathogens, the World Health Organization compiled a list of threatening bacteria. Here, the list indicates human pathogens that belong to the normal human microbiota but cause infection in the human body, including *Enterococcus faecium* (*E. faecium*), *Streptococcus pneumonia* (*S. pneumonia*), and *Staphylococcus aureus* (*S. aureus*) [5]. These infectious pathogens, which belong to Gram-positive bacteria, are major contributors to surgical site infections that constitute over 20% of hospital infections, posing significant risks to patients [6]. Furthermore, they impede the rapid treatment of tumors by reducing the efficacy of chemotherapy drugs [7].

*E. faecium* is one of the species closely associated with human infections among *Enterococci*, which are anaerobic bacteria that naturally inhabit the gastrointestinal tracts of animals and humans [8]. The medical view especially focuses on resistance to glycopeptides, with the confirmation of *E. faecium* resistance to vancomycin [9]. Yang et al. investigated that the pathogenicity genes of *E. faecium* consisted of hyaluronidase (*hyl*) and enterococcal surface protein (*esp*) within the *E. faecium* isolates. According to research findings, the *esp* gene (74.1%) is most closely related to disease outbreaks [10]. Moreover, a previous study on *E. faecium* found a significant association between the *esp* gene and immunity to other antibiotics (e.g., ampicillin, ciprofloxacin, and imipenem) [11]. Similarly, *S. aureus* is a Gram-positive bacterium with high pathogenicity, mostly in the nasal mucosa and skin of the human body. Major symptoms involving osteomyelitis, pneumonia, and gastroenteritis, even with serious manifestations, can appear because of their susceptibility to infection [12]. The immunity evasion of *S. aureus* by opportunistic infections is a key characteristic closely associated with bacterial virulence and immune-modulating factors [13]. In particular, the catalase A gene (*katA*) of *S. aureus* modulates antibiotic resistance and oxidative stress [14]. Thus, *S. aureus* can easily adapt to other aerobic environments during infections owing to the high H_2_O_2_ resistance of the *katA* gene [15]. Therefore, in this study, the *esp* gene in *E. faecium* and the *katA* gene in *S. aureus* were selected as target DNA for the recognition of key pathogenic microorganisms and the rapid treatment of hospitalized patients.

For the early detection of these foodborne pathogens, it is important to determine a suitable method for nucleic acid amplification technique (NAAT) which can be available to identify the genetic information associated with the infection. Within several amplification technologies, the polymerase chain reaction (PCR) has high accuracy and specificity and is most commonly utilized [16]. However, the PCR requires adequate temperatures for each denaturation, annealing, and extension step, as well as specialized equipment such as a thermocycler and skilled expertise, leading to high costs and labor-intensive processes [17]. Based on other studies, the loop-mediated isothermal amplification (LAMP) assay has the capability to be employed instead of PCR as a point-of-care testing (POCT) platform owing to its isothermal temperature. Furthermore, the use of 4 to 6 primers in LAMP enables higher specificity among the isothermal amplifications such as rolling circle amplification (RCA), helicase-dependent amplification (HDA), cross-priming amplification (CPA), and nucleic acid sequence-based amplification (NASBA) [18,19].

Various techniques have been developed for the endpoint detection of LAMP, such as fluorescence [20], chemiluminescence [21], electrochemical detection [22], and colorimetric detection [23]. In particular, colorimetric detection provides simple and fast visual detection of amplicons and is recognized as an effective platform for POCT [24]. Among the diverse colorimetric detection methods, pH-based colorimetry enables the immediate observation of reactions with the naked eye, dispensing the use of complex instrumentation. However, this method has a limitation in experimental settings owing to the need for additional low-buffered reagents, which is related to the effective reactivity of pH-sensitive dyes [25]. As an alternative to pH-based colorimetry, visual detection using metal nanoparticles such as gold nanoparticles (AuNPs) and silver nanoparticles (AgNPs) has been actively investigated.

One of the major methods for amplicon detection based on AuNPs involves the use of DNA-functionalized AuNPs technology. For instance, Zhao et al. demonstrated the generation of stable DNA/AuNP complexes through the facile conjugation of thiol-DNA by covering the AuNP surface with a stable layer using a mononucleotide adsorption method for AuNP-based detection in molecular diagnosis. However, the DNA/AuNP approach requires an additional surface functionalization step and a lengthy incubation time [26]. Moreover, DNA-functionalized AuNPs exhibit limited performance due to their significant influence on the reaction efficiency depending on their size, necessitating meticulous size control processes and reliance on specific targets [27]. In contrast, colorimetric detection based on the real-time fabrication of metal nanoparticles via the reduction of metal ions allows for stabilized outcomes and visual readouts enabled by remarkable shifts in plasmonic characteristics owing to particle aggregation or etching, irrespective of the target sequence [28]. The development of various biological and chemical agents capable of reducing the formation of metal nanoparticles has attracted considerable attention [29]. Furthermore, research on the use of biocompatible and biodegradable materials as reducing agents for the chemical reduction of metal NPs is underway to be developed [30].

**Figure 1:**
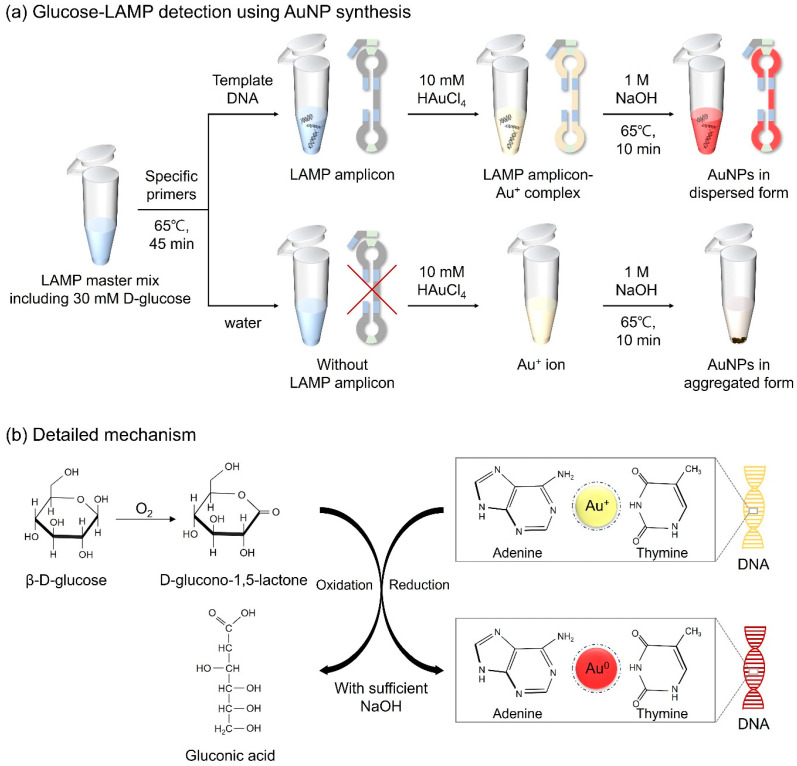
Schematic diagram of (**a**) the strategy for detecting LAMP amplicons via AuNP-based colorimetry in a microtube and (**b**) the detailed mechanism of the redox reaction.

D-glucose, a monosaccharide belonging to the carbohydrate class, is a highly significant biological compound that constitutes cells in animals, plants, fungi, and protozoans [31]. In a previous study, D-glucose, in the presence of sodium hydroxide (NaOH), was confirmed to possess both reducing and capping abilities for the formation of AuNPs from gold ions, thereby enhancing the particle stability [32]. Thus, D-glucose was selected as the reducing agent for the molecular diagnosis of the target DNA through AuNP-based colorimetric detection. Here, we designed a detection assay in which LAMP amplicon-Au^+^ complexes were reduced to LAMP amplicon-Au^0^ through the oxidation of D-glucose induced by NaOH, resulting in red coloration. Furthermore, we optimized unique LAMP protocols that combined D-glucose with LAMP reagents prior to amplification, with constant reactivity under the conditions of the LAMP assay for further colorimetric detection. The detailed mechanism is illustrated in Figure 1. Based on the experimental results, we developed AuNP-based colorimetry by optimizing the concentrations of D-glucose, NaOH, and HAuCl_4_ for the effective diagnosis of pathogens. This introduced method eliminates the need for laborious sample preparation and extensive processing time, making it suitable for use in POCT platforms for the detection of infectious microorganisms. Furthermore, since the detection results can be readily distinguished by the naked eye, it is expected to provide an affordable method that does not require expensive equipment or specialist methodologies.

## 2. Materials and Methods

### 2.1. Experimental Supplies

Gold(III) chloride trihydrate (HAuCl_4_∙3H_2_O), D-glucose, and sodium hydroxide (NaOH) were purchased by Sigma-Aldrich (St. Louis, MI, USA). dNTP mix was supplied by BioFact (Daejeon, Republic of Korea). The LAMP reagents composed of 10× isothermal amplification buffer, 100 mM MgSO_4_, and Bst 2.0 WarmStart DNA polymerase were supplied by New England Biolabs (Ipswich, MA, USA). The 100-bp DNA marker, agarose powder, and ethidium bromide dye (Loading STAR) were obtained from Takara (Kusatsu, Japan), BioShop (Burlington, ON, Canada), and Dyne Bio (Seongnam, Republic of Korea), respectively. The Wizard Genomic DNA Extraction Kit was supplied by Promega (Madison, WI, USA). The *E. faecium* spp. (ATCC: BAA-2127) and *S. aureus* plasmids were supplied by ATCC and Addgene (Watertown, MA, USA), respectively. An ultraviolet illuminator for capturing the agarose gel was supplied by Korea LabTech (Incheon, Republic of Korea).

### 2.2. Bacterial Cultures and DNA Purification

The *E. faecium* spp. was incubated with 5 mL of Luria-Bertani broth at 37 °C, and 200 rpm for 17 h in the incubator. After the growth of *E. faecium*, the bacterial culture solution (1 mL) was purified using a Wizard Genomic DNA Extraction Kit to acquire genomic DNA (gDNA) of the *esp* gene. The purity of the gDNA was determined by measuring the ultraviolet (UV) absorbance ratio at 260/280 nm using a NanoDrop™ spectrophotometer.

### 2.3. Primer Design

Five sets of primers consisting of two outer primers (F3 and B3), one loop primer (LB), and two inner primers (FIP and BIP) were designed using the PrimerExplorer V5 software for the amplification of *E. faecium* and *S. aureus*. The specific primer sequences used for the amplification of *E. faecium esp* and *S. aureus katA* are listed in Table 1.

### 2.4. D-Glucose-Based LAMP Assay (Glucose-LAMP Assay)

To employ the colorimetric detection technology based on the fabrication of AuNPs, D-glucose, a reducing agent, was assayed with LAMP reagents before the LAMP reaction. A certain quantity of D-glucose did not inhibit amplification even when mixed with LAMP reagents and maintained reactivity during the heating process for LAMP. A total of 25 μL of the Glucose-LAMP mixture was comprised of 10 μL of 30 mM D-glucose (12 mM), 3.5 μL of 10 mM dNTPs (1.4 mM), 2.5 μL of the 10× buffer, 0.5 μL of 80 μM FIP and BIP (1.6 μM), 0.5 μL of 10 μM F3 and B3 (0.2 μM), 0.5 μL of 40 μM LB (0.8 μM), 1.5 μL of 100 mM MgSO_4_ (6 mM), 0.5 μL of Bst 2.0 DNA polymerase (8 units/mL), 1 μL of target DNA solution, and 3.5 μL of deionized water. To prepare the target DNA solution, *S. aureus* plasmid was purchased from Addgene. *E. faecium* was obtained by bacterial culture and subsequent DNA purification. The mixture was then heated at 65 °C for 45 min. For the negative control, an equal volume of water was added to the mixture instead of the template DNA solution. The LAMP amplicons were visualized via gel electrophoresis and colorimetric detection based on the formation of AuNPs.

### 2.5. Colorimetric Detection for Glucose-LAMP Assay

For fabricating AuNPs, the LAMP amplicons were combined with gold chloride trihydrate (HAuCl_4_∙3H_2_O, 2.5 mM) and NaOH (0.5 M), and kept at 65 °C for 10 min. In the presence of LAMP amplicons, Au^+^ ions can bind to the base of the target DNA. DNA provides physical support to maintain its dispersed form when the gold ions are reduced to AuNPs. Through the formation of AuNPs, samples displayed a red color. The overall colorimetric detection mechanism is shown in Figure 1. In the case of negative controls, because of the absence of LAMP amplicons, AuNPs failed to sustain a dispersed form, causing the particles to aggregate and form a precipitate. Immediate discrimination between the negative and positive samples was discernible with the naked eye, facilitating the specific detection of foodborne pathogens.

### 2.6. Qualitative Test on Paper

Based on the introduced colorimetry mechanism, the results were represented by the difference in the degree of precipitation between the negative and positive samples. However, AuNPs can also be produced in negative samples, similar to positive samples, owing to the presence of a reducing agent and gold ions. The filter paper was used to detect the aggregates effectively. For distinct observations, colorimetric samples (5 μL) were dropped onto various membrane filter paper discs. Whatman filter paper (GE Healthcare Life Sciences, Shanghai, China), positively charged nylon membranes (Roche, Basel, Switzerland), glass microfibers (GF5 grade, CHMLAB, Terrassa, Spain), glass fibers (GF-C grade, Sigma-Aldrich, USA), and nitrocellulose membrane (Thermo Fisher Scientific, Waltham, MA, USA) were selected for testing. Each piece of paper had a diameter of 4 mm.

### 2.7. Quantification of Colorimetric Detection

The color intensity of the AuNPs was measured using the ImageJ 1.54i software to quantify the degree of aggregation. Furthermore, to clearly distinguish the aggregates in the images, the Wolfram Mathematica 14.0 program (https://www.wolfram.com/mathematica/, accessed on 20 November 2023) was used to analyze the data by representing the samples in a binary form. The quantified data are represented in bar graphs to illustrate the color intensity factor.

### 2.8. Sensitivity and Specificity Tests

A 10-fold serial dilution of the target DNA was conducted to measure the limit of detection (LOD) of *S. aureus katA* and *E. faecium esp* genes and to examine sensitivity. Following the introduced method, the results were successfully verified through gel electrophoresis and colorimetric detection. For the specificity test, two targets were cross-validated by amplification using primers that did not match each target and were detected using AuNP-based colorimetric detection.

## 3. Results and Discussion

### 3.1. Selection of Optimum Paper Discs

**Figure 2:**
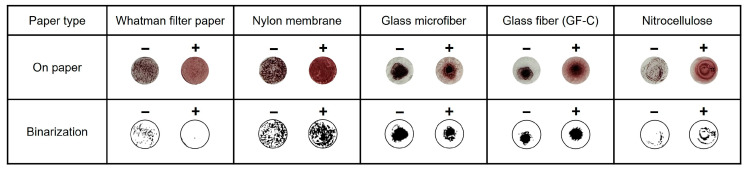
Various paper discs are used for detecting AuNPs aggregation.

According to the mechanism of the introduced method, the distinction between the negative and positive samples is based on the difference in the presence of precipitates. In the microtubes, additional time was required for the precipitate to settle in the negative samples. Hence, various paper discs that allow immediate observation of the precipitate were prepared for testing, which is effective in indicating the difference. For the AuNP-based colorimetric detection, 5 μL of 10 mM HAuCl_4_, 10 μL of 1 M NaOH, and 5 μL of LAMP amplicons were mixed in the microtube. After heating at 65 °C for 10 min, 5 μL of the mixture was dropped on prepared paper discs (4 mm). A Whatman filter paper, positively charged nylon membranes, glass microfibers, glass fibers, and nitrocellulose membranes were tested. The results were then presented in binary form for analyzing the quantification of aggregation. As shown in Figure 2, the positive samples precipitated in the same manner as the negative samples in all types of paper discs except for the Whatman filter paper. Therefore, based on these results, the Whatman filter paper was selected for further colorimetric detection to easily verify the difference in the aggregation levels between negative and positive samples.

### 3.2. Glucose-LAMP Assay

**Figure 3:**
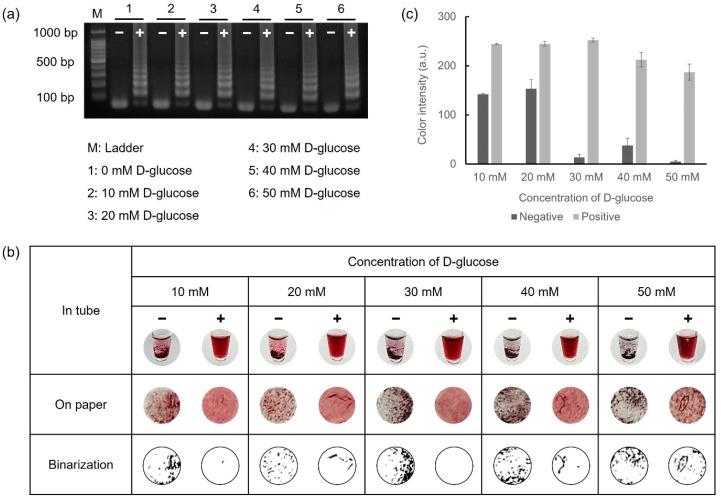
Results for optimizing D-glucose concentration via gel electrophoresis and colorimetric detection. (**a**) Glucose-LAMP assay was performed with 10 mM to 50 mM of D-glucose at 65 °C for 45 min. (**b**) Colorimetric detection was performed in microtubes and on paper discs. (**c**) The color intensity was analyzed using the ImageJ program as a graph.

D-glucose, a critical reagent for reducing gold ions, was mixed with the LAMP reagents prior to performing LAMP. The D-glucose retained consistent reactivity even in the heating process at 65 °C for 45 min during the amplification. Furthermore, the LAMP reagents preserved their reactivity for LAMP, irrespective of the D-glucose concentration. To observe the color variations based on the D-glucose concentration, 10^8^ CFU/mL of *E. faecium* was used as the target DNA. Reactions with 10–50 mM D-glucose were verified via gel electrophoresis and colorimetric detection under conditions where the concentrations of HAuCl_4_ (10 mM) and NaOH (1 M) were fixed. For colorimetric detection, 5 μL of 10 mM HAuCl_4_ and 10 μL of 1 M NaOH were mixed with 5 μL of LAMP amplicons. The overall results are shown in Figure 3. Colorimetric detection was visually observed both in tubes and on the Whatman filter paper discs, as shown in Figure 3b. Furthermore, precipitation on the paper discs was exhibited in binary form using Wolfram Mathematica 14.0 software, allowing for direct visual confirmation. As shown in Figure 3c, the positive samples exhibited nearly identical color intensities at all concentrations. However, for the negative samples, a relatively high color intensity was observed at concentrations below 20 mM, whereas an obvious aggregation difference between the negative and positive samples was evident at concentrations above 30 mM. Based on these results, 30 mM of D-glucose was adopted as the optimal condition for the colorimetric detection of foodborne pathogens.

### 3.3. Optimization of NaOH Concentration

**Figure 4:**
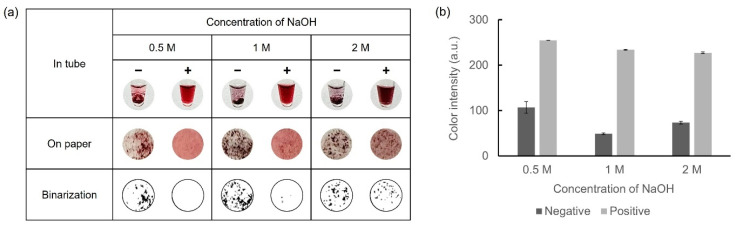
Results showing the optimization of the NaOH concentration for colorimetric detection of LAMP amplicons. (**a**) Colorimetric detection was performed in microtubes and on paper discs. The results were expressed in binary form for detailed observation of the precipitate. (**b**) A color intensity graph is shown using ImageJ software.

Owing to the requirement of sufficient NaOH for AuNP synthesis, the concentrations of NaOH within a range of 0.5–2 M were selected. Glucose-LAMP assay was performed at 65 °C for 45 min with 30 mM of D-glucose to prepare the LAMP amplicons. 10 mM HAuCl_4_ was mixed with 5 μL of LAMP amplicons and 10 μL of each concentration of NaOH. The results of the colorimetric detection and color intensity graphs are presented in Figure 4. With 0.5 M NaOH, an insufficient amount of AuNPs was not aggregated, resulting in a relatively red supernatant in the negative samples. Conversely, with 1 M and 2 M NaOH, the negative samples exhibited a dark color owing to the aggregation of a sufficient number of AuNPs. However, with 2 M NaOH in the positive samples, excessive AuNPs were produced and aggregated, resulting in dark coloration and precipitation. Consequently, 1 M NaOH was considered the optimal concentration for facilitating the oxidation of D-glucose.

### 3.4. Optimization of HAuCl_4_ Concentration

**Figure 5:**
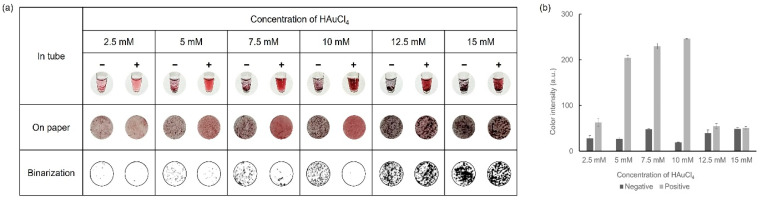
Results showing the optimization process of HAuCl_4_ concentration via (**a**) colorimetric detection and (**b**) color intensity in a graph.

Another crucial factor in the colorimetric detection of the formation level of AuNPs was HAuCl_4_, which was tested at various concentrations to achieve optimal results. With the concentrations of D-glucose (30 mM) and NaOH (1 M), which were fixed at the optimal values previously determined, colorimetric detection was performed using various concentrations of HAuCl_4_ ranging from 2.5–15 mM. Observations were performed for each concentration, both in tubes and on paper discs, and the results are shown in Figure 5. As the concentration of HAuCl_4_ increased, a stronger color was observed while excessive precipitation occurred at concentrations above 12.5 mM HAuCl_4_. When HAuCl_4_ concentrations were 7.5 mM and 10 mM HAuCl_4_, distinct color differences, as well as aggregation formation between the negative and positive samples, were shown. In particular, more aggregates were formed in the negative sample when using 10 mM HAuCl_4_. Through these optimization processes, we ultimately determined that 30 mM D-glucose, 10 mM HAuCl_4_, and 1 M NaOH were the ideal concentrations for the visual colorimetric detection of infectious pathogens.

### 3.5. Specificity Test for Diagnosis of Foodborne Pathogens

**Figure 6:**
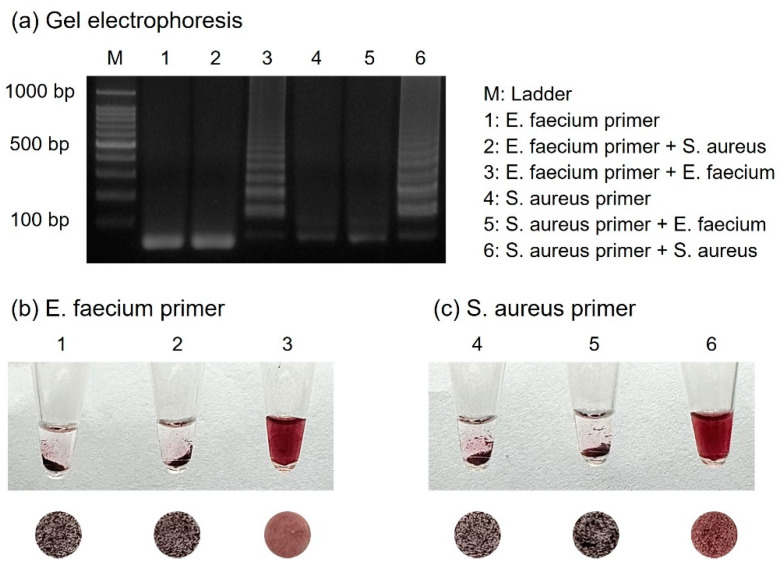
(**a**) Results of gel electrophoresis showing specificity test for *E. faecium* and *S. aureus* when performed with each set of primers. The results show the colorimetric detection when using (**b**) *E. faecium* primers and (**c**) *S. aureus* primers.

For the specific detection of numerous foodborne pathogens, primers should be designed to amplify only their respective targets. Two primer sets were prepared for the detection of *E. faecium* and *S. aureus*, and each template DNA was subjected to cross-reactivity with the other primers. As shown in Figure 6, *E. faecium* was identified only in the presence of *E. faecium* primers. Similarly, *S. aureus* was identified only when *S. aureus* primers were present. Samples with successful amplification exhibited a dispersed form upon colorimetric detection, indicating the presence of suitable primers. Conversely, each sample using primers that did not match the target DNA failed to amplify, resulting in aggregated AuNPs due to the absence of amplicons.

### 3.6. Sensitivity Test for Diagnosis of Foodborne Pathogens

**Figure 7:**
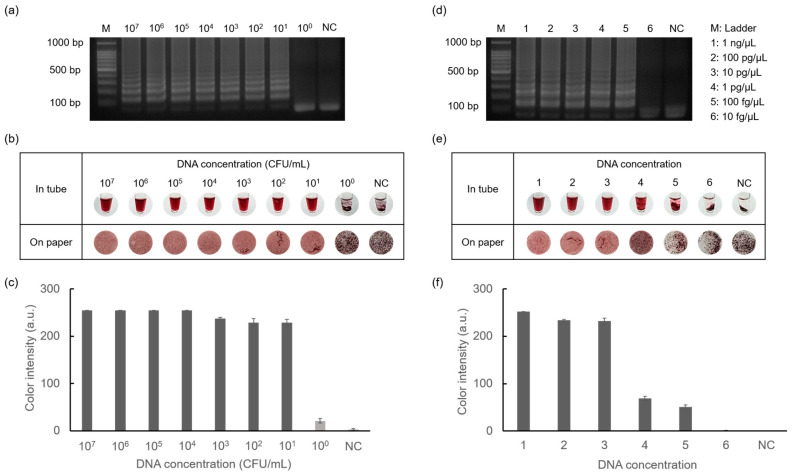
Results showing sensitivity tests for detecting *E. faecium* and *S. aureus* performed by gel electrophoresis and colorimetric detection. (**a**) Results of amplification of *E. faecium* in the range of 10^7^ CFU/mL to 10^0^ CFU/mL concentrations and negative control. (**b**) Results of AuNP-based colorimetry and (**c**) color intensity graph of *E. faecium*. Similarly, the results showed amplification of *S. aureus* in the range of 1 ng/μL to 10 fg/μL concentrations and negative control (**d**–**f**).

The sensitivity of the detection of *E. faecium* and *S. aureus* was tested using the introduced method, as shown in Figure 7. Starting from the highest concentrations of *E. faecium* (10^8^ CFU/mL) and *S. aureus* (10 ng/μL), each target was serially diluted 10-fold until reaching 10^0^ CFU/mL and 10 fg/μL, respectively. As shown in Figure 7a,d, ladder-like bands were observed for *E. faecium* at concentrations as low as 10^1^ CFU/mL and 100 fg/μL for *S. aureus*. This indicated successful amplification, even at low DNA concentrations. Colorimetry results were obtained by gel electrophoresis, confirming the sensitivity of the method for the detection of foodborne pathogens. Furthermore, colorimetric detection was additionally applied to Whatman filter paper discs, demonstrating more specific aggregation differences depending on the DNA concentration. Subsequently, data quantification allowed for the visualization of color intensity in graph form using ImageJ software. These results demonstrate that D-glucose-mediated AuNP-based colorimetry exhibits high accuracy and stability.

There are numerous types of colorimetric detection methods for the diagnosis of LAMP amplicons, including pH-sensitive colorimetry and magnetogenosensor assays [23,33]. Compared to these methods, colorimetric detection based on the formation of metal nanoparticles from metal ions requires no additional process for the preparation of experimental materials and can be completed within 10 min to accurately determine the endpoint results. Furthermore, since the AuNP-based colorimetry requires the isothermal condition at 65 °C, it can be expected to be utilized in a POCT platform for the simple recognition of foodborne pathogens within 1 h. Recently, various techniques have been introduced for the detection of infectious pathogens. However, the majority of these techniques are complex and expensive. Significantly, the chemicals involved in the detection process of the introduced method are inexpensive and readily available, making it cost-effective. Table 2 shows a list of other colorimetric detection methods compared to this study, verifying the simplicity and high sensitivity of AuNP-based colorimetry.

## 4. Conclusions

The colorimetric detection of LAMP amplicons using AuNPs has been performed using various techniques. However, a real-time AuNP synthesis-based colorimetric detection method using D-glucose as the reducing agent has not been evaluated. In this study, we optimized essential conditions such as the concentrations of HAuCl_4_, NaOH, and D-glucose in the AuNP-based detection assay through extensive experimentation. Significantly, we simplified the experimental process by pre-mixing D-glucose with LAMP reagents to avoid aerosol contamination and false-positive results. Using this method, we successfully determined the specificity and sensitivity of two critical infectious pathogens, *E. faecium* and *S. aureus*. Leveraging this colorimetric detection method, we achieved high sensitivity for *E. faecium* with an LOD of 10^1^ CFU/mL and *S. aureus* with an LOD of 100 fg/μL. Owing to its high stability, reproducibility, and accuracy, the D-glucose-mediated AuNP-based strategy is expected to pave the way for the rapid detection of infectious pathogens and the application of novel and innovative approaches to the POCT system in the development of molecular diagnosis, regardless of various on-site environmental conditions and settings.

## Figures and Tables

**Table 1:** The list of primer sequences used for the amplification of *esp* and *katA* genes.

Target Genes	Primers	Primer Sequences (5′–3′)
*esp* gene (*E. faecium*)	F3	CCAGAACACTTATGGAACAG
	B3	GTTGGGCTTTGTGACCTG
	FIP	CGTGTCTCCGCTCTCTTCTTTTTATTTGCAAGATATTGATGGTG
	BIP	ATCGGGAAACCTGAATTAGAAGAAGAACTCGTGGATGAATACTTTC
	LB	TGATGTTGACACAACAGTTAAGGG
*katA* gene (*S. aureus*)	F3	ACGATCTTAATGTCAGATAGAGG
	B3	TTGAGATGAATCGCGATCT
	FIP	ACACGTTCACCAGAATCATTATACAGATTCCTAAAGATTTGCGTCAC
	BIP	AATTCCATTTTAGAACGCAACAAGGTGCTATAATTTCAGCAGCTACT
	LB	GTGTGTGAACCGAACCCATGCA

**Table 2:** List of diagnoses of various target DNA with colorimetric detection to compare the efficiency of the introduced method.

Target DNA	Sensitivity	Process Time	Reference
*E. faecium*	10^2^ CFU/mL	50 min	[34]
SARS-CoV-2	400 copies/µL	30 min	[35]
*E. coli*	10^3^ CFU/mL	100 min	[36]
*S. aureus*	10^1^ CFU/mL	60 min	[37]
*S. pneumoniae*	10^7^ copies/µL	90 min	[38]
*S. aureus*	100 fg/µL	60 min	This work
*E. faecium*	10^1^ CFU/mL	60 min	This work

## Data Availability

Data are contained in this article.
